# Understanding Fibroblasts in Order to Comprehend the Osteopathic Treatment of the Fascia

**DOI:** 10.1155/2015/860934

**Published:** 2015-08-19

**Authors:** Bruno Bordoni, Emiliano Zanier

**Affiliations:** ^1^Don Carlo Gnocchi IRCCS, Department of Cardiology, IRCCS S. Maria Nascente Don Carlo Gnocchi Foundation, Via Capecelatro 66, Milano, Italy; ^2^School CRESO, Osteopathic Centre for Research and Studies, Falconara Marittima, Castellanza, Italy; ^3^EdiAcademy, Viale Forlanini 65, Milano, Italy

## Abstract

The osteopathic treatment of the fascia involves several techniques, each aimed at allowing the various layers of the connective system to slide over each other, improving the responses of the afferents in case of dysfunction. However, before becoming acquainted with a method, one must be aware of the structure and function of the tissue that needs treating, in order to not only better understand the manual approach, but also make a more conscious choice of the therapeutic technique to employ, in order to adjust the treatment to the specific needs of the patient. This paper examines the current literature regarding the function and structure of the fascial system and its foundation, that is, the fibroblasts. These connective cells have many properties, including the ability to contract and to communicate with one another. They play a key role in the transmission of the tension produced by the muscles and in the management of the interstitial fluids. They are a source of nociceptive and proprioceptive information as well, which is useful for proper functioning of the body system. Therefore, the fibroblasts are an invaluable instrument, essential to the understanding of the therapeutic effects of osteopathic treatment. Scientific research should make greater efforts to better understand their functioning and relationships.

## 1. Introduction: Definition of Fascia

**Figure 1:**
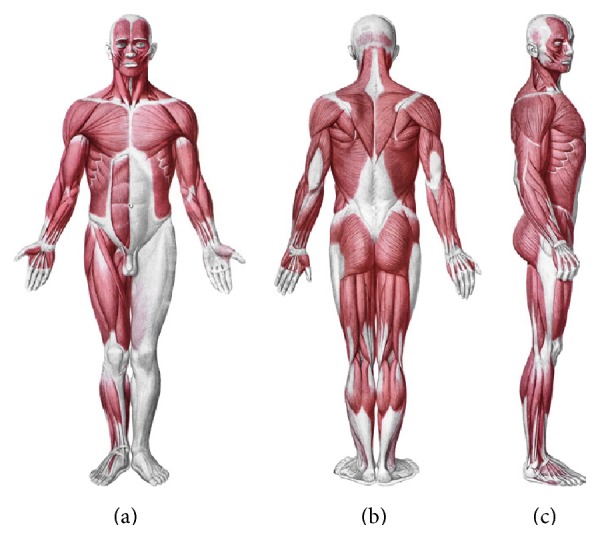
The myofascial system. Anatomical interactions between fascia and muscle. Shape and arrangement of the muscles on the ventral surface (a), dorsal (b), and lateral (c) of the human body. Reproduced with permission Anastasi et al. AA VV, Anatomia dell'uomo, 4 ed, Edi.ermes, Milano [Human Anatomy].

The osteopathic treatment of the fascia involves several techniques, each aimed at allowing the various layers of the connective system to slide over each other, improving the responses of the afferents in case of dysfunction. Every body structure is wrapped in the connective tissue or fascia, creating a structural continuity that gives form and function to every tissue and organ [1–4]. The human body must be considered as a functional unit, where every area is in communication with another through the fascial continuum, consequently originating perfect tensegritive equilibrium [5]. Medical literature does not suggest a sole definition of fascia, because it varies in terms of thickness, function, composition, and direction depending on its location. The fascial tissue is equally distributed throughout the entire body, enveloping, interacting with and permeating blood vessels, nerves, viscera, meninges, bones, and muscles, creating various layers at different depths, and forming a tridimensional metabolic and mechanical matrix [6, 7]. The fascia becomes an organ that can affect an individual's health [8]. Awareness of its functions and of the areas it controls becomes significant within a more general perspective concerning the patient's wellness and health (Figure 1).

From an embryological perspective, the fascial system originates in the mesoderm, although according to some authors this connective network can be partially found in the neural crests (ectoderm), with particular reference to the cranial and cervical area [8–10]. The most external layer is denominated subcutaneous fascia or loose (areolar) connective fascia [7, 11]. This layer is made up of several levels, each with variable amounts of fibroblasts (i.e., connective cells) arranged in a disorderly manner and soaked in a gelatinous substance known as extracellular matrix, where numerous molecules (i.e., glycosaminoglycans, proteoglycans, and polysaccharides such as hyaluronic acid) can be found [3, 12]. The superficial fascia is composed of irregularly arranged collagen fibers that are markedly different from the regularly arranged collagen fibers recognizable in tendons, ligaments, or aponeurotic sheets [1]. The fibroblasts produce a collagen subunit, tropocollagen, which is used to construct larger collagenous aggregates to form collagen fiber. This superficial layer is not located exclusively under the derma, but it permeates the entire body, enveloping the organs and forming the stroma, the neurovascular branches, and the different fascia of the muscle districts, finally resting on the deep fascia [13–15]. The superficial fascia is made up of different layers, whose formation facilitates the sliding of one layer over another, as of the structures enveloped or in contact with the aforesaid fascia [12–15]. The number of layers of the superficial fascia and the amount of substances they contain depend on the quantity of fat, the gender, and the body area concerned [12, 13]. The superficial fascia is rich in water, arranged in liquid crystals [16]. The various layers communicate by a microvacuolar system, which is in turn composed of the same structures of the superficial fascia; it is a microscopic web, concerning vessels and nerves, in varying directions, and is highly deformable [11]. According to some texts, within the superficial fascia there is a vascular network independent of the lymphatic and blood pathways. It is called the Bonghan duct system and supposedly eases communication among all body areas [17–21]. This system is composed of the same substance forming the superficial fascia [9].

The deep fascia is the last connective layer before coming in contact with the somatic structure (i.e., bones and muscles) and the visceral and vascular systems. It is characterized by various levels of loose connective tissue [3, 22]. Its vascular and lymphatic system is well developed, with numerous corpuscles in charge of proprioception, particularly the Ruffini and Pacini corpuscles [22]. It is a less extendable fibrous layer, with collagen fibers arranged more regularly, thick and parallel to each other; it is rich in hyaluronic acid [7, 22]. More precisely, the deep fascia is characterized by several layers of parallel collagen fibers and each layer is separated from the next by a layer of loose connective tissue.

According to some authors, the fascial layer enveloping the organs is a serous fascia, but in fact it is the prolongation of the deep fascia [1, 23]. All fascial layers contain a variable amount of fibroblasts with the ability to contract, known as myofibroblasts. They contain a type of actin similar to the one traceable in the muscles of the digestive system, that is, alpha-smooth muscle actin [6]. Scientific research has proven that the fascial continuum is innervated by the autonomic sympathetic system [6].

## 2. Actions and Functions: Fibroblasts and Fascial Continuum

The uniqueness of the fascial continuum consists in its composition, thanks to the many different structures that cooperate with each other, guaranteeing the human body health and integrity. From an osteopathic perspective, if the fascia is the philosophy of the body, meaning that each body region is connected to another, osteopathy is the philosophy of medicine, meaning that the entire human body must work in harmony [1].

The fibroblasts are the foundation of the fascial system [24]. They play a fundamental role in conveying tension and can dynamically affect mechanical tension, rapidly remodeling their cytoskeletons, without turning into myofibroblasts; this mechanism can occur in a few moments, as the result of a physiological change in length sustained by the fascia [25]. When the fascial tissue lengthens, the fibroblasts flatten themselves and expand, increasing their area of action. In this way, the fascia can sustain the tension without difficulty, as the flattening and lengthening of the fibroblasts result in a slighter and more sustainable strain. This phenomenon mainly occurs within the loose or areolar connective tissue, preventing the thickest and deepest layer from unnecessary strain. The fibroblast's cytoskeleton is made of microtubules, namely, actin filaments and intermediate filaments; specifically, the flexibility of actin enables a more rapid adaptation of the fibroblasts in the presence of compressive forces, due to the lengthening of the fascia [26].

The fibroblasts in the fascial system have different mechanical and metabolic behavior. The fibroblasts housed in the most superficial layer and in the various layers of loose connective tissue behave differently, with respect to those located in the thickest and deepest layer. If the mechanical information is present for only a short period of time, any morphological variation is reversible, and the cytoskeleton of the fibroblast can be restored to its original state [2, 27]. Any variation in the form of a cell due to tension enhances a series of metabolic responses that perfectly reflect the nature, direction, and duration of the tension itself. This mechanism is known as mechanotransduction; tension is the “language” of cells [28, 29]. The adaptation of the cells (and consequently their survival and that of all the systems) depends on the cells' ability to adjust themselves and change their form [15]. In this mechanometabolic scenario, a fibroblast is not a mere passive element. In fact, it not only undergoes a morphological variation due to tensional information, but can also activate itself in order to perceive the tensional level surrounding it, so as to be constantly updated and ready to adapt in real time. Through the structures connected to the extracellular matrix, that is, the integrin proteins, mechanical forces are conveyed into the fibroblast, then propagating to the nucleus and originating some metabolic responses; the fibroblasts can also carry the integrin complex, so as to assess the surrounding mechanical environment [29]. This is probably a strategy aiming at the survival of the fascial continuum, given its fundamental importance for the entire body system. The ability to successfully distribute the tension that daily acts on the human body, for instance, while walking or sitting for hours, preserves the blood vessels and the nerve pathways [15, 29]. A nonphysiological mechanical state can alter this active/passive relationship, decreasing the ability of the fibroblast to adapt and to work properly [29].

Every fibroblast is potentially aware of the functional state of the one close to it, as well as those distant from it, ensuring the fascial and mechanical continuity [14]. Between two cells there are gap junctions, made up of two cells known as connexons, which create continuity. They consist in six identical (homomeric) or different (heteromeric) proteins, called connexins [30]. These junctional structures facilitate the conveyance of mechanical information, as well as of small molecules and electrical activity [30, 31]. Communication is possible with distant cells and not necessarily with those close to one another [14]. Recent research has revealed the existence of tunnels of nanotubes that differ from the connexons, because they allow the continuation of the membrane even when it is far from the original cell, has an irregular direction, and can reach many centimeters in length [32]. These nanotubes are characterized by a contractile structure, thanks to F-actin and VA myosin [32]. This characteristic supposedly eases a rapid transmission of metabolic and electrical information, as the communication between cells takes place just in a few minutes; these connections do not appear to be permanent but transient [33].

After undergoing a mechanotransductive stimulus, with consequent epigenetic events, a fibroblast communicates its information to near and distant cells, confirming the need for and the existence of the fascial continuum. An interesting hypothesis explaining the reason for the creation of nanotubes is the possibility of conveying vital information from a sick cell to a healthy one, moving its metabolic and electric storage, and preserving its function [33]. Similarly, it could be a mechanism that easily repairs a fibroblast that does not function properly.

The mechanical environment can be directly affected by the fibroblasts, thanks to their ability to control and modulate the extracellular matrix, which indirectly determines the function of the different systems dealing with the fascial continuum [14, 15]. The fibroblasts and the extracellular matrix are closely related to each other through some contact proteins, such as the integrins; the amount of elements composing the extracellular matrix varies depending on tensional information [29]. This relationship can be noxious when there is excessive tension, developing fibrosis, or it can remain functional, increasing the sustainability of the mechanical forces, through a correct viscous and elastic environment. This mechanism is due to enzymes and growth factors directly produced by the fibroblasts, which degrade or stimulate the synthesis of numerous extracellular components, and to immune substances, creating an inflammatory environment; the change produced by the fibroblasts takes just a few hours to occur [10, 15, 24, 34–36]. The enzymes produced by the fibroblasts are the matrix metalloproteinases (MMPs), whose function is degradation, and the tissue inhibitors of metalloproteinases (TIMPs), which inhibit the MMPs; the balance among all these enzymes is vitally important in order to ensure successful tissue repair [37]. The fibroblasts secrete many growth factors and molecular subclasses, such as the connective tissue growth factor (CTGF/CCN2), the transforming growth factor-*β* (TGF-*β*), and the fibroblast growth factor (FGF): key molecules that are essential in order to preserve a proper metabolic environment [37–40]. Multiple cytokines, chemokines, and prostanoids are synthesized. The fibroblasts play a significant active role in stimulating inflammatory processes, because they are responsible for a suitable cleaning, repair, and replacement of the elements of the fascial continuum that have been and are affected by traumas resulting from daily use [4, 10, 35]. These fibroblastic characteristics aim to ensure a correct performance in managing the tension, perceived and produced.

A further action ascribable to the fibroblasts, and affecting the tension of the fascial system, is regulating the pressure of fluids and the flow of liquids that permeate the fascia. When the fibroblast undergoes a strain, the water inside is expelled toward the extracellular matrix; this mechanism is made possible by the numerous negative charges of glycosaminoglycans attracting the water, with partial and temporary loss of contact between the integrins and the matrix [25]. As soon as the tensional information is complete, the fibroblasts return to their original size and reestablish contact with the extracellular matrix through the integrins, reabsorbing the water [25]. The movement of the water outside the fibroblasts increases the stiffness of the fascia, affecting the responses of the fascial continuum in the presence of mechanical stress [25].

The fibroblasts can manage the temperature of the extracellular matrix, thanks to the renewal of its components, allowing a physiological and functional sliding of the different layers of the fascial continuum [12]. Hyaluronic acid acts as a lubricant, and its quantity affects the temperature among the fibroblasts. According to research, some specific fibroblastic cells, called fasciacytes, supposedly produce hyaluronic acid, ensuring an optimal viscous and elastic environment [12].

The fascial system can be plausibly considered a memory organ, because it not only registers the functions of the structures it surrounds and connects, but also memorizes any function or information arriving and departing from the same structures [41]. The fibroblasts and the connective tissue remember the morphological variations they have undergone, and this probably influences the tension expressed [4, 42].

The fibroblasts contain receptors for the growth hormone (GH), and depending on the levels of growth hormone circulating they can secrete insulin-like growth factors (IGFs) [24]. The IGF is a molecule characterized by multiple activities, such as facilitating tissue repair and influencing the metabolic environment.

There is a close relationship between the endocannabinoid or endorphin system and the fibroblasts. The cannabinoid receptor or CB1 is mainly housed in the nervous system, but it can be found in the fascial system and in the fibroblasts as well, particularly near the neuromuscular junction [43]. This relationship is believed to better manage any inflammation and pain information originating in the fascial tissue, as the fascia undergoes continuous remodeling during the day [43, 44].

The cells of the fibroblasts directly affect contractile tissue repair. They secrete different soluble substances, such as insulin-like growth factors (IGFs), fibroblast growth factors (FGFs), hepatocyte growth factor (HGF), interleukins (ILs), and nitric oxide (NO), as response to mechanical information undergone by the muscles;they control the differentiation of the myoblasts or precursor muscle cells, orientating their epigenetic response [44, 45].

**Figure 2:**
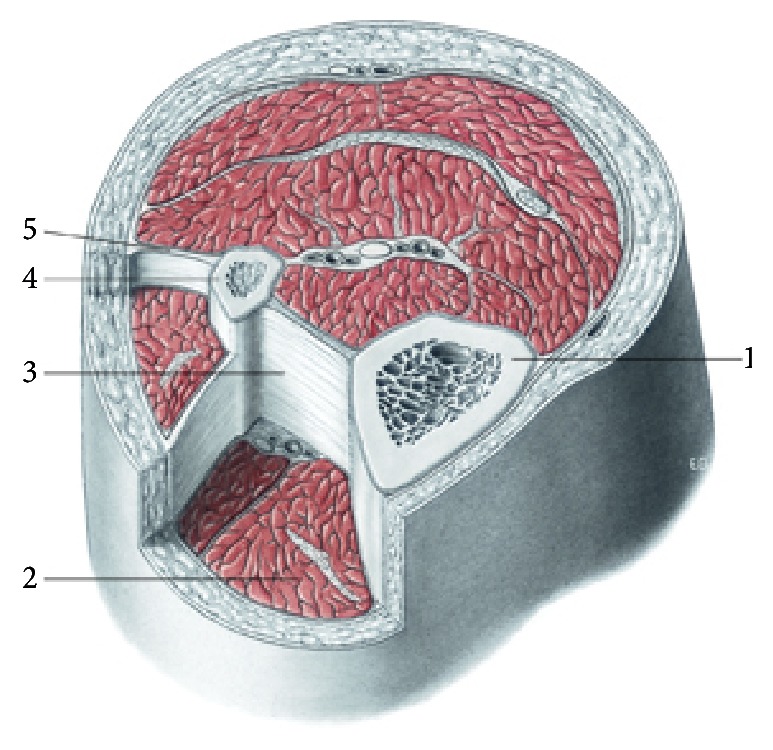
The muscular fascial system. Transverse section at the level of the upper third of the leg. 1, tibia; 2, muscular compartment; 3, interosseous membrane; 4, fibula; 5, intermuscular septum. All tissues are enveloped by fascial continuum. Reproduced with permission Anastasi et al. AA VV, Anatomia dell'uomo, 4 ed, Edi.ermes, Milano [Human Anatomy].

The fascial continuum is essential for transmitting the muscle force, for a correct motor coordination, and for preserving the organs in their site: the fascia is a vital instrument that enables the individual to communicate and live independently. The transmission of the force is ensured by the fascial integrity, which is expressed by the motor activity produced; the tension produced by the sarcomeres results in muscle activity, using the various layers of the contractile districts (epimysium, perimysium, and endomysium), with different directions and speed [6, 11, 46, 47] (Figure 2).

The connective tissue can control the orientation of the muscle fibers, so as to reflect the vector of the force's direction and to render the transition of the tension more fluid and ergonomic [46]. The fascial system is rich in proprioceptors, particularly the Ruffini and the Pacini corpuscles, mostly in the areas of transition between the articulation and the fascia, and between the fascia and the muscular tissue, blending with the receptors of these structures [6, 8]. The fascial continuum can be considered a sense organ of human mechanics, which affects daily postural patterns [6, 8, 48].

During embryo ontogenesis, the myofascial system is complete in 45 days, reflecting the physiology of an adult [49]. We can assume that the fascia works as an instrument controlling the kinetic chains, in order to preserve their completion through its tensegrity, respecting the ontogenetic impulse of the movements demonstrated by Blechschmidt's studies [49]. A characteristic of the fascia that is supposed to affect the expression of movement is the ability to carry electrical activity. The collagen proteins have semiconductive, piezoelectric, and photoconductive properties, in vitro; therefore, the fascial continuum is theorized to produce and distribute electrical activity through the extracellular matrix [6, 16, 42]. The fascial system is supposed to be electrically activated, similar to some neuronal patterns, because of different embryological derivations of the fibroblasts located in the connective tissue [10]. This electric current is thought to be influenced by different elements, such as heat, mechanical stress, and light [42]. The information affecting the fascial electrical current is supposed to influence the behavior of the many structures involved with the fascia, such as posture [7]. However, further studies are needed.

According to research carried out on live subjects or in vitro, a characteristic of the connective tissue and of fibroblasts is the ability to emit faint biophotons, which are electromagnetic field quanta; this property is typical of all the living cells in humans [50, 51]. This emission is supposed to be related to the metabolic activity of cells and to be regulated by the circadian rhythm, which means a major quantity of biophotons in the morning, decreasing in the evening [52]. The connective tissue is also thought to store these biophotons and to control their emission, depending on the body area involved; for instance, in the head and in the hands there is a greater level of emission. The release of biophotons is believed to be affected by temperature; in fact, a greater level of emission has been proven at about 37°C [53]. It is still unknown whether this activity is a waste product of metabolism or, on the contrary, another means of communication between cell and cell, as some authors assert [54].

The continuity of the fascial system is vitally important for all the organs it surrounds and correlates, because it guarantees their correct functionality, in addition to metabolic and hormonal responses of these structures [7, 8, 42].

A further important role played by the fascial system is the management of liquids, such as lymph and blood. In fact, the fascia activates the flow of lymph and blood toward the different structures meant for containing the liquids; this activity is attributable to the innate contractile property of the fascia but also to different pressure gradients generated by the various fascial layers, which compel the liquids to flow [7, 18–21]. The flowing of liquids is important for cellular health [7]. According to some authors, the fascial system and the fibroblasts have a sort of “memory” mechanism of the dysfunction. Even the reactivity of the spine and its consequent mobility are supposedly due to a neurofascial memory [55]. According to the memory theory, the direction of the fibroblasts in transmitting the force and their persistence in maintaining such a tensional vector define tensional memory. Further studies are needed to know more about memory of this tissue.

## 3. Osteopathic Fascial Techniques

Fibroblasts are the foundations of the fascial system, and to know their behavior and reaction to the manipulative techniques, it becomes essential to improve the technique itself and to better understand the symptom picture of the patient. Currently, we still know little on the adaptation of fibroblasts, in the presence of a stimulus like a manual osteopathic treatment.

Osteopathic techniques aim to release fascial restrictions, to mobilize tight ligaments, and to drain congested lymphatics [56]. The purpose of these therapies and treatments is to alter the mechanical properties of fascia, such as density, stiffness, and viscosity, so that the fascia can more readily adapt to physical stresses [57]. In fact, some osteopathic physicians and manual therapists report local tissue release after the application of a slow manual force to tight fascial areas; these reports have been explained as a breaking of fascial cross-links, a transition from gel to sol state in the extracellular matrix, and other passive viscoelastic changes of fasciae [57]. The fascial osteopathic technique is the application of a low load, long duration stretch into the myofascial complex, intended to restore the optimal length of this complex [58]. The practitioner palpates the fascial restriction and the pressure is applied directly to the skin, into the direction of restriction, until resistance (the tissue barrier) is felt [6]. Once found, the collagenous barrier is engaged for 90–120 seconds, without sliding over the skin or forcing the tissue, until the fascia complex starts to yield and a sensation of softening is achieved [6]. We do not know the exact scientific reasons for this fascial release, despite the many studies showing that a fascial osteopathic treatment is useful in many clinical conditions [6]. In vitro studies demonstrate how the osteopathic techniques can influence the metabolic behavior of fibroblasts, such as proliferation and inflammatory response [59]. The myofascial techniques practiced by manual operators utilize a very similar approach to indirect techniques. Myofascial release is a widely employed direct manual medicine treatment which utilizes specifically guided mechanical forces to manipulate and reduce myofascial restrictions of various somatic dysfunctions [60]. It is proved that by applying this method, fibroblasts are able to change their orientation and probably their mechanical behavior. Another possible explanation comes from a study that employed the fascial techniques but not resembling the indirect techniques. An improved sliding of the various fascial layers would allow resetting the afferent of the free nerve endings, resulting in physiologic response of the efferent [61]. Further studies are expected in order to understand better the behavior of fibroblasts, as a result of indirect and fascial techniques, so as to choose the best osteopathic approach to well-being of the patient.

## 4. Conclusion

The fibroblasts represent the foundation of the fascial system, a structure of connective tissue that covers and affects every body area. These cells have many properties, including the ability to contract themselves and to communicate with one another. They play a key role in the transmission of the tension produced by the muscles and in the management of the interstitial fluids. They are a source of nociceptive and proprioceptive information as well, which is useful for proper functioning of the body system. Despite the significant number of studies and research on the aforesaid cells, there is still much to understand and to investigate. We hope that this paper may be useful to the health professionals who deal with the treatment of the fascial continuum and that it becomes a stimulus for researchers in order to implement our competence on this extraordinary tissue.
